# Seasonal Variations in Notification of Active Tuberculosis Cases in China, 2005–2012

**DOI:** 10.1371/journal.pone.0068102

**Published:** 2013-07-10

**Authors:** Xin-Xu Li, Li-Xia Wang, Hui Zhang, Xin Du, Shi-Wen Jiang, Tao Shen, Yan-Ping Zhang, Guang Zeng

**Affiliations:** 1 National Center for Tuberculosis Control and Prevention, Chinese Center for Disease Control and Prevention, Beijing, P. R. China; 2 Chinese Field Epidemiology Training Program, Chinese Center for Disease Control and Prevention, Beijing, P. R. China; University of Witwatersrand, South Africa

## Abstract

**Background:**

Although seasonal variation in tuberculosis (TB) incidence has been described in many countries, it remains unknown in China.

**Methods:**

A time series decomposition analysis (X-12-ARIMA) was performed to examine the seasonal variation in active TB cases nationwide from 2005 through 2012 in China. Seasonal amplitude was calculated for the evaluation of TB seasonal variation.

**Results:**

A total of 7.78 million active TB cases were reported over a period of 8 years. A spring peak (April) was observed with seasonal amplitude of 46.3%, compared with the winter trough (February). Most cases in provinces with subtropical and tropical monsoon climate showed lower amplitudes than those in temperate continental, plateau and mountain climate regions. The magnitude of seasonality varied inversely with annual average temperature, r (95% CI) = -0.71 (-0.79, -0.61). The seasonal amplitudes were 56.7, 60.5, 40.6, 46.4 and 50.9% for patients aged ≤14, 15–24, 25–44, 45–64, and ≥65 years, respectively. Students demonstrated greater seasonal amplitude than peasants, migrant workers and workers (115.3% vs. 43.5, 41.6 and 48.1%). Patients with pulmonary TB had lower amplitude compared to patients with pleural and other extra-pulmonary TB (EPTB) (45.9% vs. 52.0 and 56.3%). Relapse cases with sputum smear positive TB (SS+ TB) had significantly higher seasonal amplitude compared to new cases with sputum smear positive TB (52.2% vs. 41.6%).

**Conclusions:**

TB is a seasonal disease in China. The peak and trough of TB transmission actually are in winter and in autumn respectively after factors of delay are removed. Higher amplitudes of TB seasonality are more likely to happen in temperate continental, plateau and mountain climate regions and regions with lower annual average temperature, and young person, students, patients with EPTB and relapse cases with SS+ TB are more likely to be affected by TB seasonality.

## Background

Previous studies conducted in India, Japan, Mongolia, Netherlands, Russia, Spain, United Kingdom, United States, etc. between 1992 and 2012 have evaluated the seasonality of presentation for suspected tuberculosis (TB), [1] TB notification and prevalence,[2]–[13] childhood TB incidence [14] and TB cases in migrants, [15] and explored correlation of seasonal variations and vitamin D status. [3], [16], [17] Several methods have been used to evaluate TB seasonality, including Fourier analysis, [7] cosinor analysis, [11], [16] sinusoidal harmonic model, [15], [18] spectral analysis, [12] seasonal autoregressive integrated moving average model (SARIMA), [7], [17] time-series decomposition method (X-11), [8] and others.

Studies conducted in countries in the northern hemisphere have identified peak months of TB notification in spring (March to May),[6]–[8], [15], [17], late spring and early summer (April to June), [10], [13] or summer (June to August), [5], [11], [18] and trough months in the fall (September to November), [7] late fall and early winter (October to December), [6], [8], [10], [13] or winter (January to February). [18] Undoubtedly, differences of TB seasonality exist among countries, which indicate that the mechanisms underlying the seasonal variation of TB are complex and multifactoral, and need intensive studies.

China’s total population was 1.37 billion in 2011 and 15% of the world’s notified cases of TB in 2010 occurred within China’s borders. [19] China has an area of 9.6 million square kilometers with various different climate zones, including temperate, tropical or subtropical, and frigid climate zones. Given this diversity, the studies for seasonality of TB notification in China will provide important evidence also for other countries.

There are few studies that have evaluated the seasonality of TB in China. A study conducted in Hong Kong demonstrated seasonality of TB cases reported in 2005, [18] and SEIR (susceptible, exposed, infected and resistant) TB models with seasonality were developed to simulate the seasonal variation of the reported cases of active TB in China. [20], [21].

The Chinese Center for Disease Control and Prevention (China CDC) has been conducting national surveillance for TB annually since 2002, leading to the establishment of the National Center for TB Control and Prevention (NCTB) in March 2002, and the National TB Information Management System (NTIMS) based on the internet in January of 2005. [22] These measures made the nationwide TB surveillance a reality and also made the research on TB seasonality possible on a nationwide scale. For this study, we utilized information on TB cases notified in the NTIMS between 2005 and 2012 to evaluate TB seasonality.

## Materials and Methods

### Data Source

The monthly notification for all forms of active TB cases from all 31 provinces from 2005 to 2012 in the mainland of China, who were directly notified to the NTIMS by all county-level TB dispensaries and monitored by NCTB immediately through the NTIMS, were analyzed.

### Time Series Analysis

Monthly raw case counts were analyzed using the X-12-ARIMA seasonal adjustment program (X-12-ARIMA program) that is an enhanced version of the X-11 Variant of the Census Method II seasonal adjustment program developed by the US Census Bureau. [23] In the X-12-ARIMA program, the original time series is decomposed into three basic components: trend-cycle, seasonal and irregular. The trend-cycle is the long-term and medium-to-long term movement of the series, including consequential turning points; the seasonal component is within-year fluctuations about the trend that recur in a very similar way in the same month or quarter each year; and the irregular component is the residual component that remains after trend-cycle and seasonal component are removed from the series.

### Statistical Steps and Methods

In the first step, the X-12-ARIMA program was applied to the raw monthly case counts. The time series of total active TB cases was decomposed into trend cycle, seasonal and irregular components. A decomposition of monthly case counts was obtained for groups of interest, according to time period, sex, age, occupation, form of TB, sputum smear test and province, which all were calculated as mean peak month, mean trough month, and annual seasonal amplitude with 95% confidence intervals (CI) for the years 2005–2012, if they had identifiable seasonality assessed by the X-12-ARIMA program. Annual seasonal amplitude was calculated from isolated seasonal factors and was defined as a fraction with the numerator being the peak-to-trough difference between the months with the highest and the lowest case counts and with the denominator being mean case counts for that year.

In the second step, the amplitudes of seasonal fluctuation were compared within groups. Local Getis-Ord Gi* was used to examine the local level of spatial autocorrelation and determine locations of clusters or hotspots. A calculated Z score of Gi* >1.96 indicates that the province and its neighboring provinces have a seasonal amplitude statistically significantly higher than other provinces, and Z score<-1.96 indicates the significantly lower seasonal amplitude. Linear correlation was used to demonstrate the correlation of amplitude of seasonal fluctuation and annual average temperature by province. The Student’s *t*-test for two independent samples was used to compare seasonal amplitudes of two subgroups. The Student-Newman-Keuls method for one way analysis of variance was used to compare all pairwise seasonal amplitudes of three or more subgroups. *P*-values <0.05 were considered statistically significant.

### Statistics Software

The Sigma Plot statistical package (SigmaPlot 11.0; Systat Software, Inc., Chicago, IL, USA) was used for graphing and correlation analysis; ArcGIS (ArcGIS 10.0; ESRI Inc., Redlands, CA, USA) was used for mapping and spatial analysis; and the Statistical Analysis System (SAS 9.2; SAS Institute Inc., Cary, NC, USA) was used for statistical comparisons of seasonal indicators within groups.

## Results

**Figure 1:**
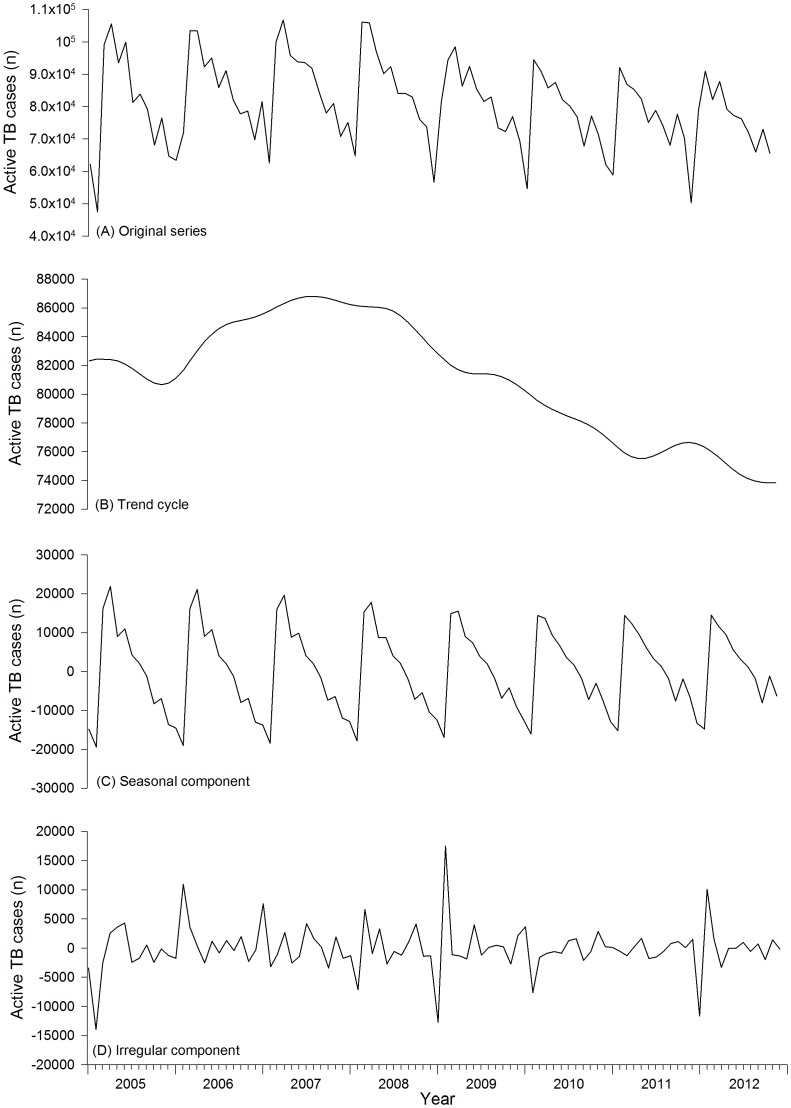
Seasonal decomposition of active tuberculosis cases per month in the mainland of China, 2005–2012. Original series (A) with trend cycle (B), seasonal component (C), and irregular component (D).

From 2005 to 2012, there were about 7.78 million active TB cases notified by all county-level TB dispensaries in the mainland of China (Table 1). Figure 1A showed original series of active TB case with the X-12-ARIMA seasonal decomposition of the isolated trend cycle (Figure 1B), seasonal (Figure 1C), and irregular (Figure 1D) components. The raw counts showed remarkably consistent seasonal fluctuation. There was a downward trend in 2005, then an upward trend from 2006 to 2007, and then a steadily decreasing trend from 2008 to 2012. From the isolated seasonal component it was found that seasonal amplitude decreased each year from 2005 to 2012.

**Table 1:** Active tuberculosis cases in the mainland of China, 2005–2012.

Group		2005	2006	2007	2008	2009	2010	2011	2012	Total
All active TB cases		96.2	101.5	104.0	103.2	98.2	93.8	91.2	89.9	778.0
Sex	Male	66.8	70.2	72.4	72.2	68.2	65.1	63.7	62.5	541.2
	Female	29.3	31.3	31.6	31.1	30.0	28.6	27.5	27.4	236.8
Age (years)	0–14	1.5	1.2	1.0	0.9	0.9	0.7	0.6	0.6	7.4
	15–24	14.9	16.6	16.8	17.4	17.1	16.3	15.7	14.5	129.3
	25–44	32.0	33.8	33.5	32.5	30.3	28.6	27.6	26.4	244.8
	45–64	29.5	30.5	32.0	32.3	31.1	30.3	30.0	30.2	245.9
	65+	18.2	19.4	20.6	20.1	18.8	17.9	17.3	18.1	150.6
Occupation	Peasant	64.9	67.2	68.8	68.2	63.7	60.7	59.4	59.2	512.0
	Migrant worker	3.1	3.7	4.1	4.1	3.8	3.9	3.1	2.5	28.3
	Worker	5.5	5.8	5.9	5.9	6.1	6.1	5.4	4.9	45.6
	Student	5.2	5.5	5.3	5.2	5.4	4.5	4.0	3.7	38.9
	Others	17.5	19.2	19.9	19.9	19.4	18.6	19.3	19.5	153.3
Form of disease	Pulmonary TB	93.1	98.3	101.0	99.9	94.8	90.7	88.1	86.8	752.6
	Pleural TB	2.3	2.4	2.1	2.3	2.6	2.5	2.5	2.5	19.1
	Other extra-pulmonary TB	0.8	0.8	0.9	1.0	0.8	0.7	0.7	0.6	6.2
Sputum smear microscopy	Positive TB	55.8	54.7	53.2	52.8	50.9	48.4	42.4	35.5	393.8
	Negative TB	35.4	42.2	46.5	46.5	43.5	42.0	45.5	51.0	352.6
Sputum smear positive TB	New case	47.3	47.2	46.4	46.4	44.9	43.0	37.7	31.5	344.4
	Relapse case	8.5	7.6	6.8	6.4	6.0	5.4	4.7	4.1	49.4

(Unit: 10 thousand).

**Figure 2:**
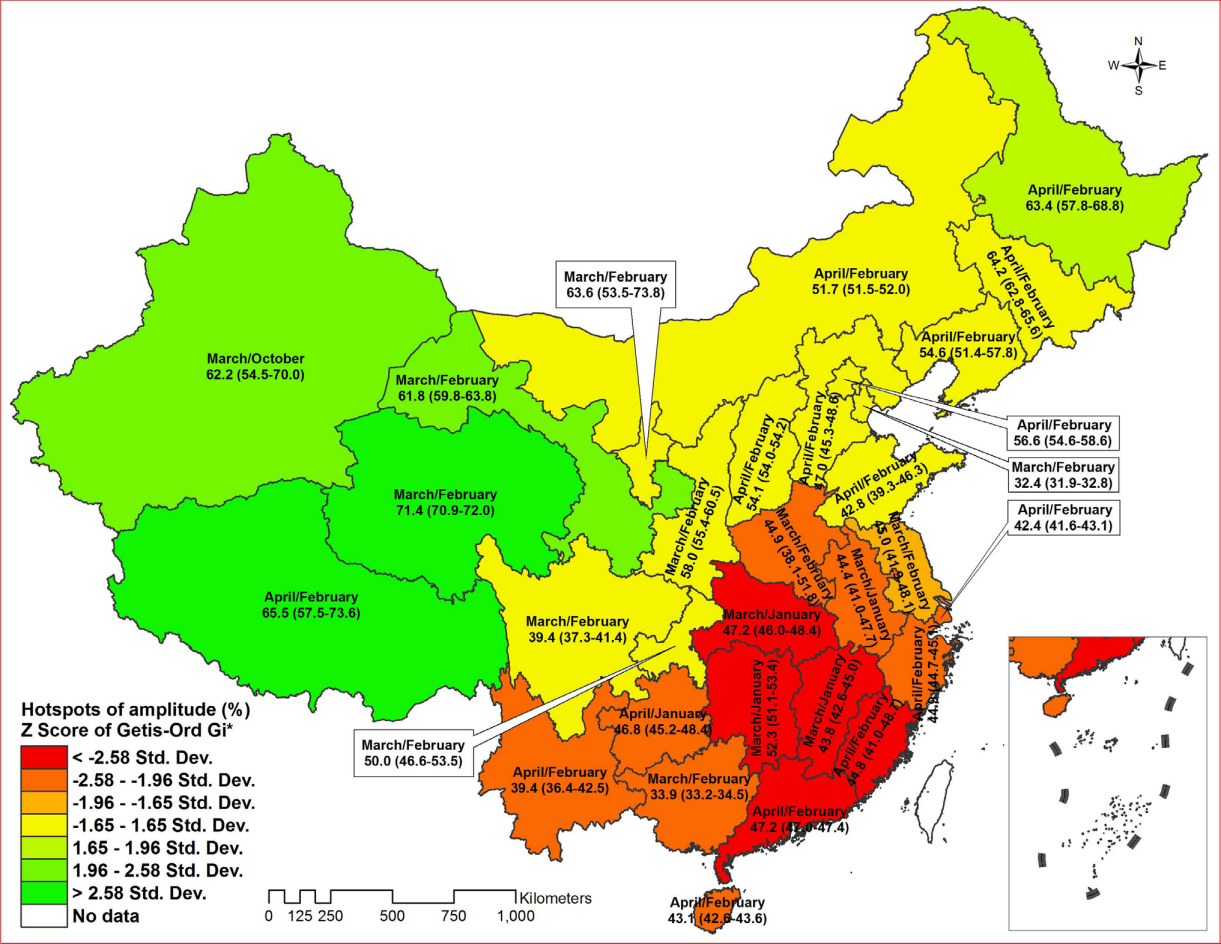
Peak/trough month, seasonal amplitude (%) (95% Confidence Interval) and hotspots of amplitude of active tuberculosis cases by province in the mainland of China, 2005–2012.

**Figure 3:**
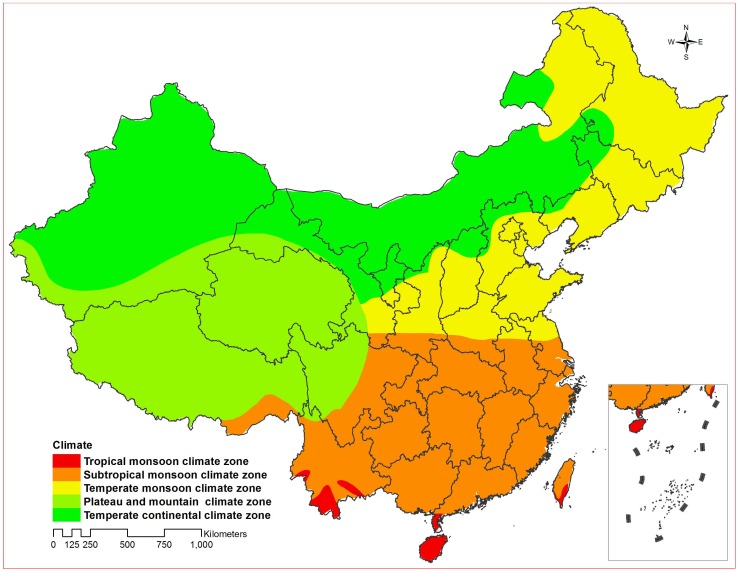
Climate zones in China.

Figure 2 illustrated peak and trough months, seasonal amplitude and clusters (hotspots) of amplitude by province in the mainland of China. Most provinces demonstrated a seasonal peak in March or April, and a trough during January or February. The result of Local Getis-Ord Gi* for spatial autocorrelation showed that there were 2 significant spatial hotspots of amplitude among 31 provinces. One hotspot with Z score >1.96 meant the area of higher amplitude and another with Z score<−1.96 meant the area of lower amplitude, which largely overlapped temperate continental, plateau and mountain climate zones and tropical and subtropical monsoon climate zones, respectively (Figure 2 and Figure 3).

**Figure 4:**
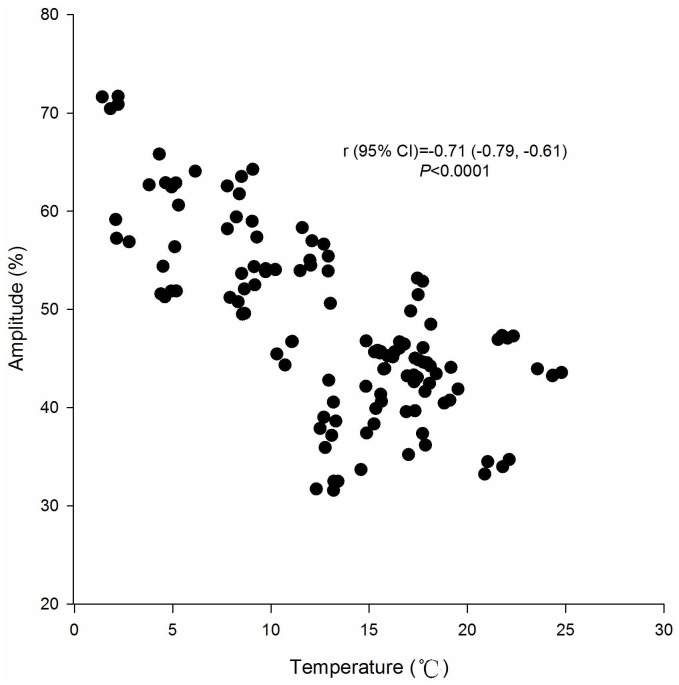
Correlation of seasonal amplitude of active tuberculosis cases and annual average temperature by province in the mainland of China, 2008–2012. Source of meteorological data: National Climate Center of China (http://ncc.cma.gov.cn/cn/). Abbreviations: CI, confidence interval.

The magnitude of seasonality was inversely correlated with temperature, with seasonal amplitude decreasing with increasing annual average temperature by province (r [95% CI] = −0.71 [−0.79, −0.61], *P*<0.0001) (Figure 4).

Table 2 summarized the peak and trough months with seasonal amplitude for subgroups of each group and the comparisons of seasonal amplitudes. An annual mean of 46.3% (95% CI, 42.3%–50.4%) more active TB cases were notified in the peak month (April) compared with the trough month (February) from 2005 to 2012. The seasonal amplitude (53.6%) of year 2005–2008 was significantly higher than that (40.6%) of year 2009–2012.There was no statistical difference in seasonal amplitude between males and females (45.0% vs. 49.5%, *P*>0.05). There were significant differences in seasonal amplitude by age, with amplitude ranging from 40.6% to 60.6% (*P*<0.05). There were significant differences in seasonal amplitude by occupation except the comparison between peasants and migrant workers, in which students had a significantly higher seasonal amplitude than workers (115.3% vs. 48.1%, *P*<0.05), peasants (115.3% vs. 43.5%, *P*<0.05) and migrant workers (115.3% vs. 41.6%, *P*<0.05), and workers had a significantly higher seasonal amplitude than peasants (48.1% vs. 43.5%, *P*<0.05) and migrant workers (48.1% vs. 41.6%, *P*<0.05). There were significant differences in seasonal amplitude by forms of TB, including pulmonary TB, pleural TB and other extra-pulmonary TB, with amplitude ranging from 45.9% to 56.3% (*P*<0.05). Patients with sputum smear negative TB had significantly higher seasonal amplitude compared to patients with sputum smear positive TB (53.0% vs. 42.9%, *P*<0.001). Relapse cases with sputum smear positive TB had significantly higher seasonal amplitude compared to new cases with sputum smear positive TB (52.2% vs. 41.6%, *P*<0.001). The peak months occurred in the spring (March, April, May) for all subgroups, with April as most common; the trough months for all subgroups occurred in February.

**Table 2:** The timing and seasonal amplitude of active tuberculosis cases in the mainland of China, 2005–2012.

Group		Peak/trough month	Seasonal amplitude (%)	SE (%)	95% CI (%)	*P* value
All active TB cases		April/February	46.3	1.6	42.3–50.4	
Period	Year 2005–2008	April/February	53.6	0.4	52.4–54.9	<0.001§
	Year 2009–2012	April/February	40.6	0.1	40.2–41.0	
Sex	Male	April/February	45.0	1.7	40.8–49.3	>0.05§
	Female	April/February	49.5	1.5	45.9–53.1	
Age (years)	0–14	May/February	56.7	0.2	56.2–57.2	<0.05¶
	15–24	April/February	60.5	1.1	57.8–63.3	
	25–44	March/February	40.6	0.8	38.6–42.5	
	45–64	March/February	46.4	1.3	43.1–49.7	
	65+	April/February	50.9	2.4	45.0–56.7	
Occupation	Peasant	March/February	43.5	1.3	40.4–46.6	<0.05¶ †
	Migrant worker	May/February	41.6	0.2	41.0–42.1	
	Worker	April/February	48.1	1.7	43.9–52.3	
	Student	April/February	115.3	0.2	114.9–115.7	
Form of disease	Pulmonary TB	April/February	45.9	1.6	41.9–49.8	<0.05¶
	Pleural TB	April/February	52.0	0.8	50.1–54.0	
	Other extra-pulmonary TB	April/February	56.3	1.2	53.4–59.3	
Sputum smear microscopy	Positive TB	March/February	42.9	1.3	39.7–46.1	<0.001§
	Negative TB	April/February	53.0	1.3	49.9–56.1	
Sputum smear positive TB	New case	March/February	41.6	1.3	38.5–44.8	<0.001§
	Relapse case	March/February	52.2	1.0	49.7–54.8	

Abbreviations: SE, standard error; CI, confidence interval.

§ Two-tailed two independent samples Student’s *t*-test for difference in seasonal amplitudes.

¶ Student-Newman-Keuls Method of one way analysis of variance for all pairwise multiple comparison among seasonal amplitudes.

† *P* value>0.05 for difference in seasonal amplitudes between subgroup of peasant and subgroup of migrant worker.

## Discussion

The X-12-ARIMA program was used to analyze the TB seasonality in this study. A study indicated that ARIMA model is the most appropriate model for forecasting seasonality pattern of seasonal diseases since it has the more relatively accuracy than models of linear regression, moving average, decomposition, Holt-Winter’s and artificial neural network. [24] Compared with ARIMA, maybe the X-12-ARIMA program is more appropriate model. The chief source of the X-12-ARIMA program is the extensive set of time series model building facilities built into the program for fitting the regARIMA models. These are regression models with ARIMA errors, in which the mean function of the time series (or its logs) is described by a linear combination of regressors, and the covariance structure of the series is that of an ARIMA process. [23].

In this study, we found TB was seasonal disease in China, whose notification figures were lowest between January and February in winter and highest between March and May in spring. The peak months were similar to what had been reported in some countries in the northern hemisphere, such as Kuwait, Mongolia, Netherlands and United States,[6]–[8], [15], [17] and earlier than other countries, such as India, Japan, Spain and United Kingdom. [4], [5], [10], [11], [13] On the contrary, the trough months were later than that of all countries noted above.

There are some delays between TB infection and notification. First, there is a long delay between onset of symptoms and initiation of notification among TB cases. The median delay was 65 days in China, [25] which also was observed in other counties. [26], [27] Additionally, maybe the Spring Festival, a Chinese traditional festival, is a special reason of delay in January or February generally, during which patients often get used to delaying health-seeking when they fall ill because health-seeking or sickness is regarded as an unlucky thing. [18] Second, there is an incubation period between infection and onset of symptoms. It was reported that the geometric mean incubation period of TB was 20.8 weeks. [28] Thus, we might infer that the peak and trough months of TB transmission are in winter and in autumn respectively, corresponding to that of TB notification in spring and in winter in China. Previous studies in many countries in the northern hemisphere also indicated that a seasonal pattern of TB with a mostly predominant peak was seen during the spring and summer seasons, which leads to assume that the risk of TB transmission appears to be the greatest during winter months. [29].

Close contact of indoor winter crowding has to be considered first for TB transmission. In winter the indoor activities are much more common than in a warm climate, which increase the probability of healthy people exposing to tubercle bacilli expelled from the infected persons in a room with closed windows for a longer period of time.[29]–[31] Maybe public transportation during the Spring Festival is another opportunity of close contact crowding in winter in China. Wherever they are, hundreds of millions of people traditionally return hometown to reunite with their family and relatives by public transportation, such as train, bus, plane or ship during this festival every year. An investigation in United States indicated the limited transmission of TB from a potentially highly infectious passenger to other persons during extended train and bus travel. [32] Therefore, we suppose that it is a good opportunity of TB transmission for a great deal of population who make a long journey in the closed coaches in winter, even though we have not direct proofs in this study.

Besides close contact of indoor crowding that is route of transmission, persons with lower Vitamin D level may be susceptible for TB infection in winter. [2], [3], [5], [7], [8], [16]–[18], [29], [31], [33] A systemic review showed that deficiency of Vitamin D impaired host immunological defense with TB infection, and serum Vitamin D concentrations gradually decreasing in autumn and winter; less sunlight exposure because of heavy clothing or indoor activities in winter influenced serum Vitamin D concentrations. [29] In this study, we found seasonal amplitude of TB were higher in temperate continental, plateau and mountain climate zones and lower in tropical and subtropical monsoon climate zones, which tended to increase while annual average temperature by province decreased in China. It was indicated that seasonal amplitudes varied with climate zone and were higher in provinces with cold temperatures for an extended part of the year. Therefore, deficiency of Vitamin D in winter due to reduced sunlight exposure may be another contributor to TB infection in China.

The seasonal pattern of TB is possibly produced by other factors. A wide variety of respiratory infectious diseases both viral and bacterial show the seasonal cycle with a winter peak, [14] which can not cause TB but may accelerate disease manifestation in patients with latent TB or increase susceptibility of individuals to infection through suppressing host immunologic capacity. [8] Seasonal variations in the nutrient intakes and the meal patterns of humans possibly affect immune system functions, and the immune system competency itself also varies periodically through the year, which may be linked with seasonal variability of TB. [29] This study was an analysis of TB surveillance data so that we had not the findings to discuss or support these alternative factors for TB seasonality, which was the main limitation of this study.

Despite the relatively large difference in TB rates, there was no significant difference in the seasonal amplitude between the two sexes in this study, which was consistent with studies conducted in United States, India and Hong Kong. [7], [8], [10], [18] However, the seasonal amplitudes of persons aged ≤24 years old were significantly higher than other persons in this study, which suggested they must have had relatively recent TB infection in winter compared with the elderly who may have been infected many years earlier. These findings were same as the study in United States. [8] The seasonal amplitudes of students were far higher than that of other population in this study maybe because seasonal TB outbreaks happened in schools. Epidemiological survey of TB outbreak in a senior high school in China suggested actually TB transmission among students happened in the closed classrooms in winter even though the outbreak was found in spring or summer. [34] Therefore, TB seasonality of students may be another evidence for recent TB transmission.

Willis, *et al*. [8] thought that TB resulting from recent transmission was more influenced by season than TB resulting from activation of latent infection but Parrinello, *et al*. [7] didn’t think so. The opinion of the latter was approved by findings in this study that seasonal amplitudes of relapse sputum smear positive TB cases were higher than that of new sputum smear positive TB cases, which may indicate activation of latent TB infection in winter. We also found that patients with extra-pulmonary TB including TB pleurisy were higher than that of patients with pulmonary TB, which also may support the opinion of activation of latent TB infection because a study of molecular epidemiology showed extra-pulmonary TB was less likely than pulmonary TB to be a result of recent transmission. [35].

This study based on a sample size of nationwide scale for 8 years with substantial information regarding patient-level and province-level characteristics was able to cast light on the potential characteristics and determinants of TB seasonality, while it was unable to illuminate the cause of seasonal variation in disease directly. We found that the seasonal amplitudes of TB notification went down during the years when the number of TB notification decreased, which suggested that there are relationships between TB notification and seasonal amplitude. We hope these findings guide the direction of future research regarding factors related to TB seasonality and targeting time of the year when intervention should be done to cut down the peak of TB transmission.
